# Femtosecond laser-induced micro/nanostructures loaded with silver nanoparticles on clear aligners prevent bacterial infection

**DOI:** 10.1186/s12903-026-08963-7

**Published:** 2026-06-23

**Authors:** Mengyu Li, Cuiyu Liu, Kaixin Wang, Cunhui Fan, Qian Yang, Yang Zhao, Yang Liu

**Affiliations:** 1 Department of Orthodontics, The Affiliated Hospital of Qingdao University, Qingdao, 266003 China; 2 School of Stomatology, Qingdao University, Qingdao, 266023 China; 3 Department of Pediatric Stomatology, Weifang Maternal and Child Health Hospital, Weifang, 261061 China

**Keywords:** Femtosecond lasers, Micro/nanostructures, Ag nanoparticles, Invisible aligners, Antibacterial

## Abstract

**Background:**

Controlling biofilm formation is crucial for maintaining aesthetically healthy teeth during clear aligner treatment. In this study, we use a femtosecond laser to prepare porous micro/nanostructures on the surface of invisible aligner for loading silver nanoparticles (AgNPs) to create an anticaries aligner.

**Methods:**

Femtosecond laser technology was used to fabricate porous micro-nanostructures on invisible aligner, after which the micro-nanostructures were loaded with green synthesized silver nanoparticles. Physical properties, chemical properties, antimicrobial properties against *S. mutans*, and biocompatibility were evaluated.

**Results:**

Laser treatment significantly increased the wettability of the aligner (water contact angle reduced from 86.5° to 38.7°, *P* < 0.05), while preserving salivary absorption rate and flexural elastic moduli. In addition, the results of in vitro antimicrobial tests showed that the laser/Ag group had a high inhibition rate of 62.89% against *Streptococcus mutans*. All treatment groups exhibited excellent biocompatibility with both mouse fibroblast (L-929) and human gingival fibroblast (HGF) cells.

**Significance:**

Our strategy provides a promising avenue for improving the antimicrobial properties of invisible orthodontic appliances. Laser treatment enhances the loading of silver nanoparticles onto aligners while having no adverse effect on the physicochemical properties of clear aligner. This innovative drug delivery platform demonstrates versatile applicability with broad material compatibility for hydrophilic nano-antimicrobials, establishing a clinically translatable framework for orthodontic infection control.

**Graphical Abstract:**

**Figure:**
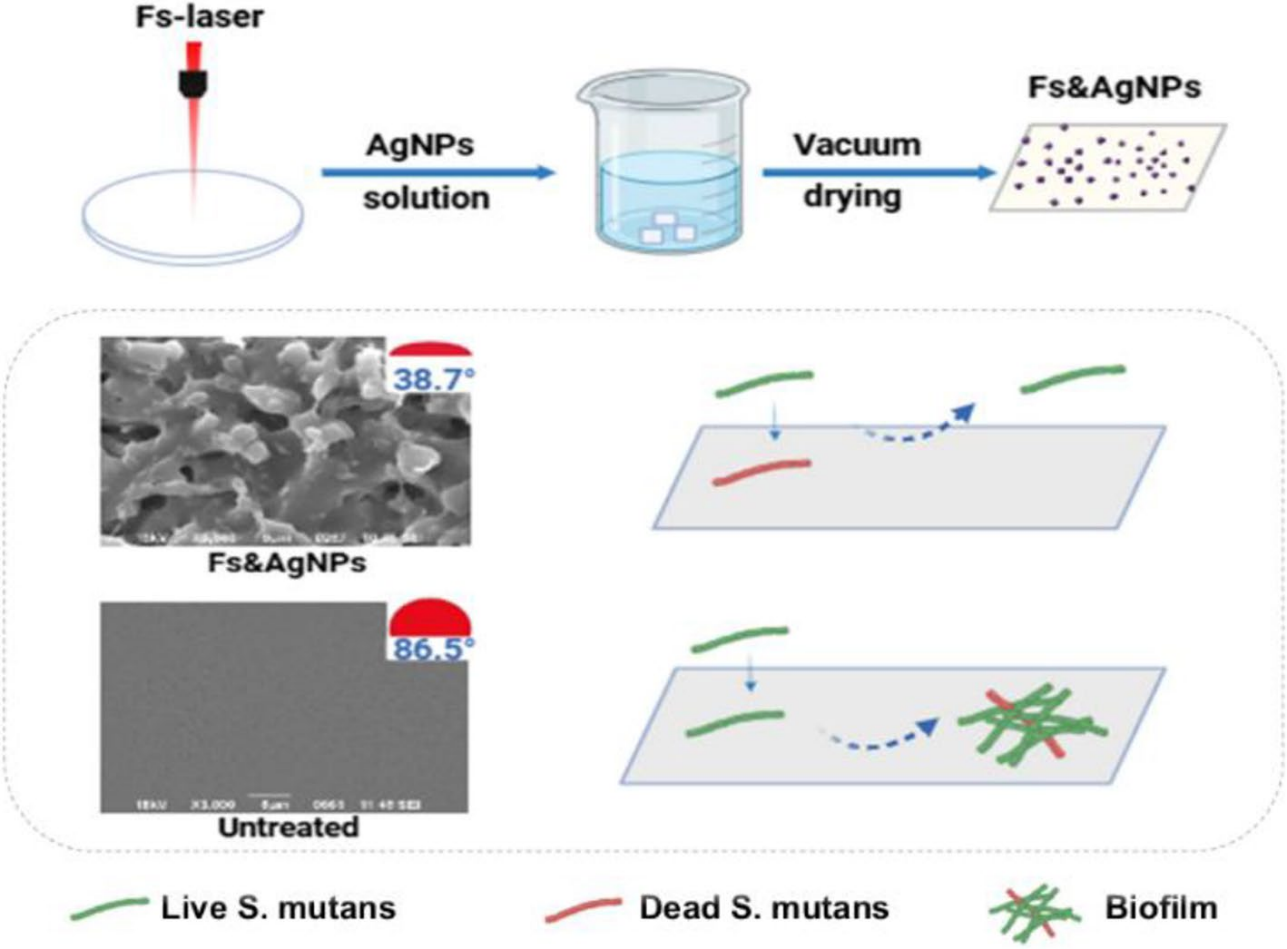

**Supplementary Information:**

The online version contains supplementary material available at 10.1186/s12903-026-08963-7.

## Introduction

Orthodontic treatment using clear aligners has become increasingly popular among patients owing to its better aesthetic appeal, comfort, and convenience compared with traditional fixed brace [1]. The demand for clear aligners has increased significantly over the past decade, with the global market size expected to increase from $3.1 billion in 2021 to $11.6 billion by 2027, at a compound annual growth rate of 13% [2]. However, when a patient's oral hygiene is not well controlled, the cariogenic plaque composed of acid-producing bacteria such as *S. mutans* accumulates between the invisible aligners and the covered tooth surface, forming a bacteria-rich, acid-producing microenvironment that is favorable for enamel demineralization [3]. In addition, the invisible aligner covers large areas of tooth surfaces for a long time, hindering the self -cleaning and buffering effects of saliva [4]. Thus, the demineralized enamel surface is difficult to remineralize, clinically manifesting as extensive and rapidly progressing white spot lesions and caries, which degrade the efficacy of the orthodontic treatment.

Various strategies have been studied for treating bacterial infections during clear-aligner treatment. The most common strategy is applying an antimicrobial coating to the surface of the aligners. Coating materials mainly include metals and metal compound materials, carbon-based materials, polymers, bioactive materials, or a combination of metal–metal or metal–nonmetal materials [5]. Currently, silver nanoparticles (AgNPs) are being intensively studied and are widely used in the oral cavity owing to their small particle size, broad-spectrum and efficient antimicrobial properties, and low cytotoxicity. Hamouda et al. used Ag/cellulose nanocomposites synthesized from marine algae with strong antimicrobial activity against multidrug-resistant bacteria isolated from the oral cavity of patients with dental caries, which can be added to toothpaste or dental fillings to prevent dental caries and improve the oral health of patients [6]. Odatsu et al. found that nanosilver coatings prepared on pure titanium healing abutment implants effectively prevented supragingival and subgingival biofilm formation and plaque build-up, which helped prevent peri-implantitis and increased the long-term success of the implants [7].

Common methods for preparing antimicrobial coatings include surface spraying, electrostatic assembly, and chemical deposition. Xie et al. successfully prepared a Au NP coating using an electrostatic self-assembly method, in which clear aligners were first treated with oxygen plasma to introduce negatively charged chemical groups and then immersed in a positively charged quaternary ammonium salt-modified Au NP solution for 12 h to efficiently and durably inhibit the formation of S. pyogenes plaques and biofilms [8]. Vas et al. used polyethyleneimine as a cross-linking agent to prepare curcumin–chitosan coatings on the surface of invisible orthodontic appliances; however, the antimicrobial effect was immersion time-dependent. For shorter immersion times (less than 5 min), the antimicrobial effect was not significant owing to poor crosslinking of the polymer-crosslinking agent, and the curcumin staining effect increased with increasing impregnation time, seriously affecting the esthetics [9]. Antimicrobial coatings have several advantages; they are a widely applied and mature technology that does not affect the thickness of the material. However, the coating process requires a long immersion time (i.e., a time-consuming process) and the resulting film is unstable and can be easily damaged in the complex oral environment, leading to loss of function [10]. Therefore, there is a need to develop a new simpler method to produce antimicrobial invisible aligners. In recent years, femtosecond lasers have been widely applied as a specialized processing technology for modifying the physical structures of material surfaces [11]. Unlike traditional physical modification methods such as spraying [12], nanoimprinting [13], plasma etching [14], electrostatic self-assembly technology [15], and hydrothermal synthesis [16], femtosecond lasers can process a wide range of materials, exhibit three-dimensional digitization and high-precision, and enable non-contact cold processing [17]. More importantly, by adjusting the laser parameters, different patterns can be fabricated on the material surface to control its wettability. To date, research progress in the laser processing of micro/nanostructures has enhanced the antimicrobial performance of various surfaces. Ma et al. used femtosecond-laser ablation combined with fluorination to fabricate honeycomb multifunctional structures that protected implants from Staphylococcus aureus and Pseudomonas aeruginosa infections [18]. Yin et al. prepared multilevel micro/nanostructures on polytetrafluoroethylene polymer surfaces by adjusting the laser parameters, resulting in materials with reduced bacterial adhesion, corrosion resistance, and less influence on the color [19]. The existing research shows that precise micro/nanostructural processing of material surfaces can be realized by the computerized adjustment of the femtosecond laser power, scanning spacing, laser wavelength, and other parameters, and the physical and chemical properties of the materials can be regulated.

Herein, we propose a novel methodology using a femtosecond laser combined with a coating of AgNPs to fabricate multifunctional 3D micro/nanostructures on invisible aligner diaphragms. First, porous micro/nanostructures were fabricated on the diaphragm surface using a femtosecond laser, followed by loading with green-synthesized AgNPs. The diaphragm surfaces were characterized and their physical, chemical, and antimicrobial properties were evaluated. Inhibiting the formation of biofilms on the surface of clear aligners by drug-carrying micro/nanostructures may help prevent bacterial infections and provide new technical support for the early prevention of caries, thereby improving the oral health of the patient and the efficacy of the orthodontic treatment.

## Materials and methods

### Materials

A clear alignment diaphragm with a thickness of 1 mm was purchased from Erkodur (Germany). Wuhan Hongtuo New Technology Co., Ltd. conducted laser treatment (China). Silver nitrate and sodium hydroxide standard solutions were provided by Shanghai Maclin Biochemical Technology Co., Ltd. (Shanghai). Tea polyphenol extract and L929 mouse fibroblast cells were purchased from Dalian Meilun Biotechnology Co., Ltd. (China). Human gingival fibroblasts (HGF) and *S. mutans* lyophilized powder (ATCC25175) were purchased from Beijing Beina Biotechnology Co., Ltd. (China).

### Laser parameters

The femtosecond laser processing parameters were selected based on previous studies [20–22] and further optimized through preliminary processing experiments (Figure S1). The parameters used in the femtosecond laser treatment included a center wavelength of 1030 nm, pulse duration of 934 fs, repetition rate of 200 kHz, scanning power of 20 W, scanning pitch of 20 µm, and scanning speed of 2000 mm/s. After laser processing, the samples were ultrasonically cleaned in anhydrous ethanol for 5 min and dried in a vacuum oven. The laser-processed aligner diaphragms are henceforth referred to as the laser group (laser), while the untreated diaphragms are the control group (control). The aligner diaphragms were cut into rectangular strips with dimensions of 100.0 mm × 10.0 mm × 1.0 mm (*l×b×h*) to meet the span requirements for the three-point bending test. For all other experiments, specimens with dimensions of 10.0 mm × 10.0 mm × 1.0 mm =(*l×b×h*) were used.

### Characterization procedures

Scanning electron microscopy (SEM; JEOL, Japan) was used to observe the micro/nanostructures. The elemental compositions of the samples were analyzed using energy-dispersive X-ray spectroscopy (EDS, Navo Nano 450), and the chemical states were determined using X-ray photoelectron spectroscopy (XPS, Thermo Scientific K-Alpha). Fourier transform infrared spectroscopy (FTIR) was used to analyze the changes in the chemical molecular composition of the samples. Scans were taken over a wavenumber range of 500–4000 cm^−1^ with a resolution of 2 cm^−1^. A total of 32 scans were averaged.

### Wettability

The water contact angle (WCA) of the samples was measured using a contact-angle meter (Kruss DSA 255, Germany) using 5 μL water droplets. Three different positions were selected for each sample; three parallel samples (n ≥ 3) were measured for each group, and the average value was taken as the final WCA.

### Mechanical properties

Three-point bending tests were conducted using a universal material testing machine to evaluate the mechanical properties of the aligner diaphragms. The samples were supported in a fixture with a span of 50 mm, deflection of 8 mm, and loading speed of 5 mm/min. The flexural modulus (E_b_, MPa) was calculated using Eq. (1), and the average value was calculated by repeating the experiment three times.

1 $${E}_{b}=\frac{{l}^{3}}{{4bh}^{3}}\times \left(\frac{\Delta p}{\Delta f}\right)$$

In this equation, ${E}_{b}$ is the flexural modulus (MPa), *l* represents the span (mm), *b* represents the width (mm), *h* represents the thickness (mm), *Δ f* represents the deflection (mm), *Δ p* represents the load force (N), and $\frac{\Delta p}{\Delta f}$ represents the slope of the load–deflection curve (N/mm).

### Saliva absorption property

The saliva absorption rate was used to evaluate the saliva absorption performance of the clear diaphragm. Before the test, all samples were dried and weighed in a vacuum drying oven, then placed in a test tube containing 2 mL of artificial saliva and placed in a 37 °C constant temperature shaking incubator, and the artificial saliva was changed daily. After 7 days of immersion, the surface was completely dried with absorbent paper and weighed. The test was repeated three times, and the saliva absorption rate was calculated according to Eq. (2), where ${W}_{0}$ and ${W}_{7}$ represent the weights before and after 7 days of immersion, respectively.

2 $$Saliva absorption rate \left(\%\right)=\frac{{W}_{7}-{W}_{0}}{{W}_{0}}\times 100\%$$

### Synthesis and loading of AgNPs

AgNPs were prepared using tea polyphenol extracts as green reducing agents: 50 mL of sterilized ultrapure water was added to a beaker, followed by 3 mL of silver nitrate standard solution at a concentration of 0.1 mol/L and 2 mL of tea polyphenol solution at a concentration of 5 mg/mL; the pH of the solution was adjusted to 9.2 with sodium hydroxide, and the solution was stirred on a magnetic stirrer at 800 rpm at room temperature until the solution turned reddish brown.

UV–visible spectrophotometer (UV–vis) was used to detect the differences in the characteristic absorption peaks of tea polyphenol solution, silver nitrate standard solution and synthesized silver nanoparticle solution at a wavelength of 200–500 nm. The EDS was used to analyze the peak position of the elemental spectrum to determine whether it contained silver. Field emission scanning electron microscope (FE-SEM) was used to observe the morphology of AgNPs. Finally, the particle size of the AgNPs was measured with a Zeta particle size analyzer.

AgNPs were loaded by simple immersion: 1 cm × 1 cm sample was placed into a screw bottle, 5 mL of silver nanoparticle solution was added and shaken for 30 min before being removed. The control group was subjected to silver nanoparticle aligner diaphragm (control/Ag group) and the laser group silver nanoparticle appliance diaphragm (laser/Ag group) were obtained by rinsing 3 times with phosphate-buffered saline (PBS) and then in a vacuum drying oven and placing them in a vacuum drying oven.

### Coating durability

To assess the stability of AgNPs within the micro/nanostructures, laser-treated diaphragms loaded with AgNPs were examined in the initial state, after immersion in artificial saliva at 37 °C for 7 days, and after standardized brushing with a soft-bristle toothbrush to simulate the mechanical friction associated with daily cleaning. After treatment, all samples were gently rinsed with deionized water to remove loosely bound residues, air-dried, sputter-coated with gold, and examined by SEM to evaluate their surface morphology.

### Antimicrobial property

In this study, *Streptococcus mutans* (*S. mutans*) was tested by contact antibacterial experiment and in vitro culture colony counting experiment. All experiments were repeated 3 times and the average value was taken.

Contact antimicrobial experiment: 2 Petri dishes containing BHI solid medium were taken, 100 µL of bacterial suspension (10^8^ CFU/mL) was added dropwise to one of the Petri dishes, and the samples were placed, then the growth inhibition of *S. mutans* was observed after incubation at 37 ℃ for 48 h in a constant temperature incubator. One drop of 20 µL bacterial suspension (10^8^ CFU/mL) was added in the center of the area divided by another petri dish, and the samples were placed on the top of the corresponding drop of bacterial suspension, so as to make full contact with the bacteria. The samples were then incubated at 37 ℃ for 48 h, and the size of the corresponding bacterial colony growth area was measured by Image J software.

In vitro culture colony counting antibacterial experiment: the sample was irradiated with ultraviolet light for 30 min, placed in a test tube, and 1 mL of BHI liquid medium and 100 μL of *S. mutans* (10^8^ CFU/mL) were piped and inoculated on different sample surfaces. A control group (without any sample) was set up in parallel. All tubes were then incubated in a shaking incubator at 37 °C for 24 h. After diluting the three groups of bacterial solutions by a certain number of times, 50 μL was coated on BHI solid medium and incubated in a 37 °C constant temperature incubator for 48 h. To evaluate the long-term antibacterial performance, samples from the three groups were immersed in 5 mL of artificial saliva at 37 °C for 7 days prior to antibacterial testing. After immersion, the samples were removed, vacuum-dried, and sterilized again by ultraviolet irradiation for 30 min. The antibacterial assay was then performed using the same procedure described above. The antibacterial rate was calculated according to Eq. (3).

3 $$A\mathrm{n}\mathrm{t}\mathrm{i}\mathrm{b}\mathrm{a}\mathrm{c}\mathrm{t}\mathrm{e}\mathrm{r}\mathrm{i}\mathrm{a}\mathrm{l} rate\left(\%\right)=\frac{{CFU}_{control}-{CFU}_{\mathrm{e}\mathrm{x}perimental}}{{CFU}_{control}}\times 100\%$$

In this equation, ${CFU}_{control}$ and ${CFU}_{experimental}$ represent the average number of colony-forming units from the control and experimental groups, respectively.

### Cytotoxicity

In this study, all samples were tested for cytotoxicity using mouse fibroblasts (L-929) and human gingival fibroblasts (HGF). Material extracts of the prepared samples were tested for extract cytotoxicity according to ISO guidelines. Mouse fibroblasts and human gingival fibroblasts were respectively inoculated in 96-well plates and incubated with different sample extracts in a cell culture incubator for 24 h at 37 °C with 5% CO_2_. The cell activity was detected by CCK-8 kit (‌Cell counting kit-8‌‌) and the OD value at 450 nm was measured spectrophotometrically as an indicator of viable cells.

In addition, the effect of the samples on the viability of both L929 cells and HGF cells was investigated using the Calcein AM/PI cell live/dead staining assay. The effects of extracts from different sample materials on the viability of both L929 cells and HGF cells were analyzed by using an inverted fluorescence microscope to observe the morphology of live cells under blue light excitation and dead cells under green light excitation.

### Statistical analysis

All data are presented as mean ± standard deviation (SD). Statistical analysis was performed using GraphPad Prism 10.12 software. The specific statistical tests were chosen based on the experimental design. Comparisons between two groups were performed using an unpaired Student's t-test. For comparisons among three groups, statistical significance was determined by one-way analysis of variance (ANOVA) followed by appropriate post-hoc tests for multiple comparisons.

## Results

### Surface properties

**Fig. 1:**
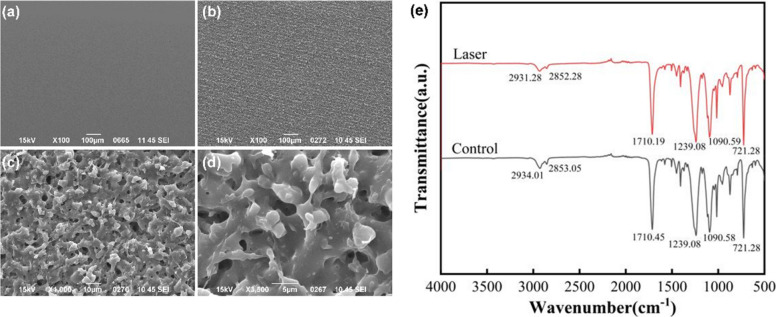
SEM images of (**a**) the control group, (**b**-**d**) the laser group. **e** FTIR profile of control and laser group

As shown in Fig. 1a, the surface of the control group is smooth and flat, whereas the laser group (Fig. 1b, c and d) has a rough topography and a porous micro/nanostructure. In addition, the low-magnification SEM images show that the layered porous microstructure is more homogeneous, with a well-defined shape and size distribution. High-magnification SEM images show longitudinal trenches between the micropores and protrusions, and nanoscale protrusions exist between the structures. The micropores have a diameter of ~ 1–5 μm, and the protrusion structures with an average diameter of 5 μm are evenly distributed over the entire surface of the processed sample.

The FTIR spectra of the two groups of appliance diaphragms (Fig. 1e) show various absorption peaks, including those related to –CH_2_ telescopic vibration peaks at 2932 cm^−1^, –CH_3_ at 2853 cm^−1^, –C = O at 1710 cm^−1^, –C–O– at 1239 cm^−1^ and 1090 cm^−1^, and para-phenylcyclohydrogen groups at 721 cm^−1^. The wavenumber positions are similar for both groups.

### Wettability

**Fig. 2:**
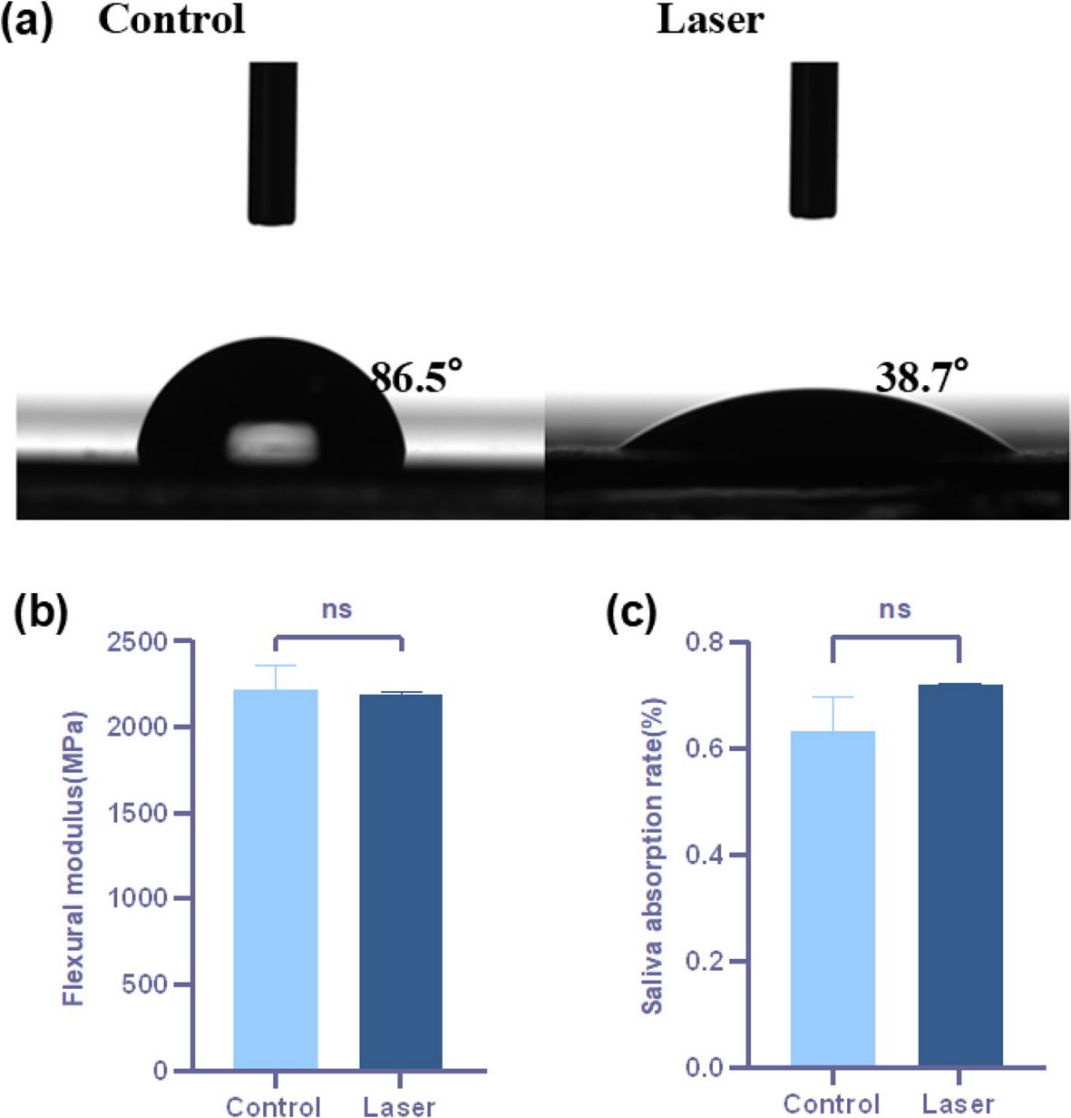
**a** Surface water contact angle. **b** The flexural modulus of elasticity. **c** Saliva absorption rate of appliance diaphragm in control and laser groups

Static WCA measurements were performed to assess the surface wettability. Figure 2 compares the recorded images of the water droplets and the WCA values for the different samples. The water droplets on the control sample formed a hemisphere with a WCA of 86.5°, while that of the laser-treated surface was significantly lower (38.7°; Fig. 2a).

### Mechanical properties

As shown in Fig. 2b, the flexural elastic modulus of the aligner diaphragms in the control group is 2214.6 ± 140.22 MPa, and that of the laser group is slightly lower (2189.97 ± 20.27 MPa), although there is no significant difference between the two groups (*P* > 0.05).

### Saliva absorption

The saliva absorption rates of the aligners in the control and laser groups are shown in Fig. 2c. The saliva absorption rate is 0.63 ± 0.06% for the control group and 0.72 ± 0.01% for the laser group. The saliva absorption rate is slightly higher after femtosecond laser treatment, but the difference between the two groups is not statistically significant (*P* > 0.05).

### Properties and loading of AgNPs

**Fig. 3:**
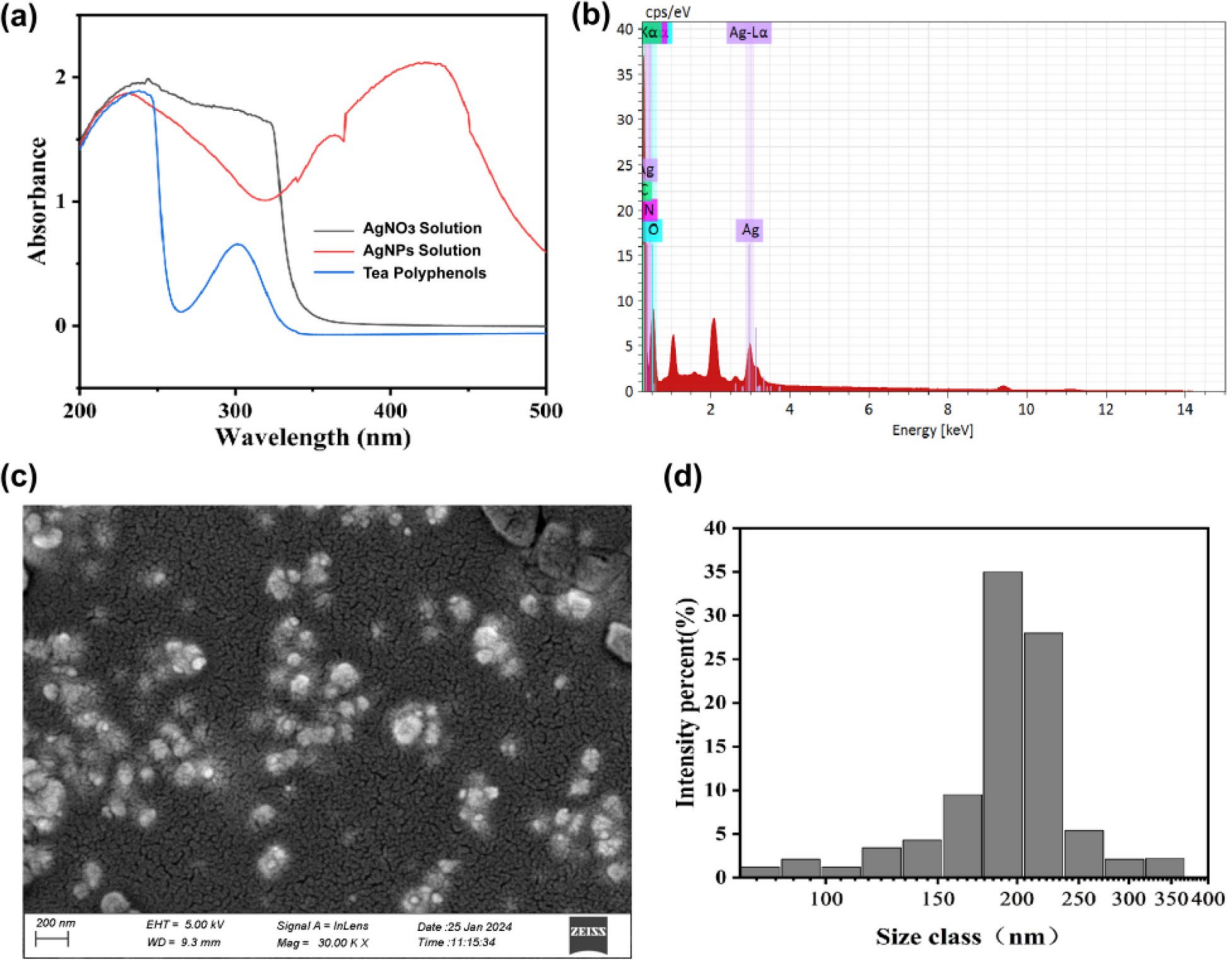
**a** UV–Vis absorption curve of tea polyphenols, silver nitrate and synthetic silver nanoparticle solution. **b** EDS scanning energy spectrum of synthetic silver nanoparticle solution. **c** FE-SEM image of synthetic silver nanoparticle solution. **d** Particle size distribution map of synthetic silver nanoparticle solution

Figure 3a shows the UV–vis absorption curves of the tea polyphenol, silver nitrate, and synthesized AgNP solutions. The use of tea polyphenols and other plant extracts as reducing and protective agents to prepare AgNPs has become a research hotspot in recent years because it offers the advantages of low cost, readily available materials, and a low environmental impact. The AgNP solution shows an absorption peak at 410–430 nm, and the EDS spectrum (Fig. 3b) shows a plasmon resonance absorption signal at 3 keV, demonstrating the successful synthesis of AgNPs. Figure 3c shows an FE-SEM image of the synthesized AgNP solution, in which the AgNPs are observed as round particles of various sizes. The results of the zeta particle size analysis in Fig. 3d indicate a range of AgNP sizes of 80–350 nm. The wide particle-size range may be related to the reaction conditions and aggregation of AgNPs.

**Fig. 4:**
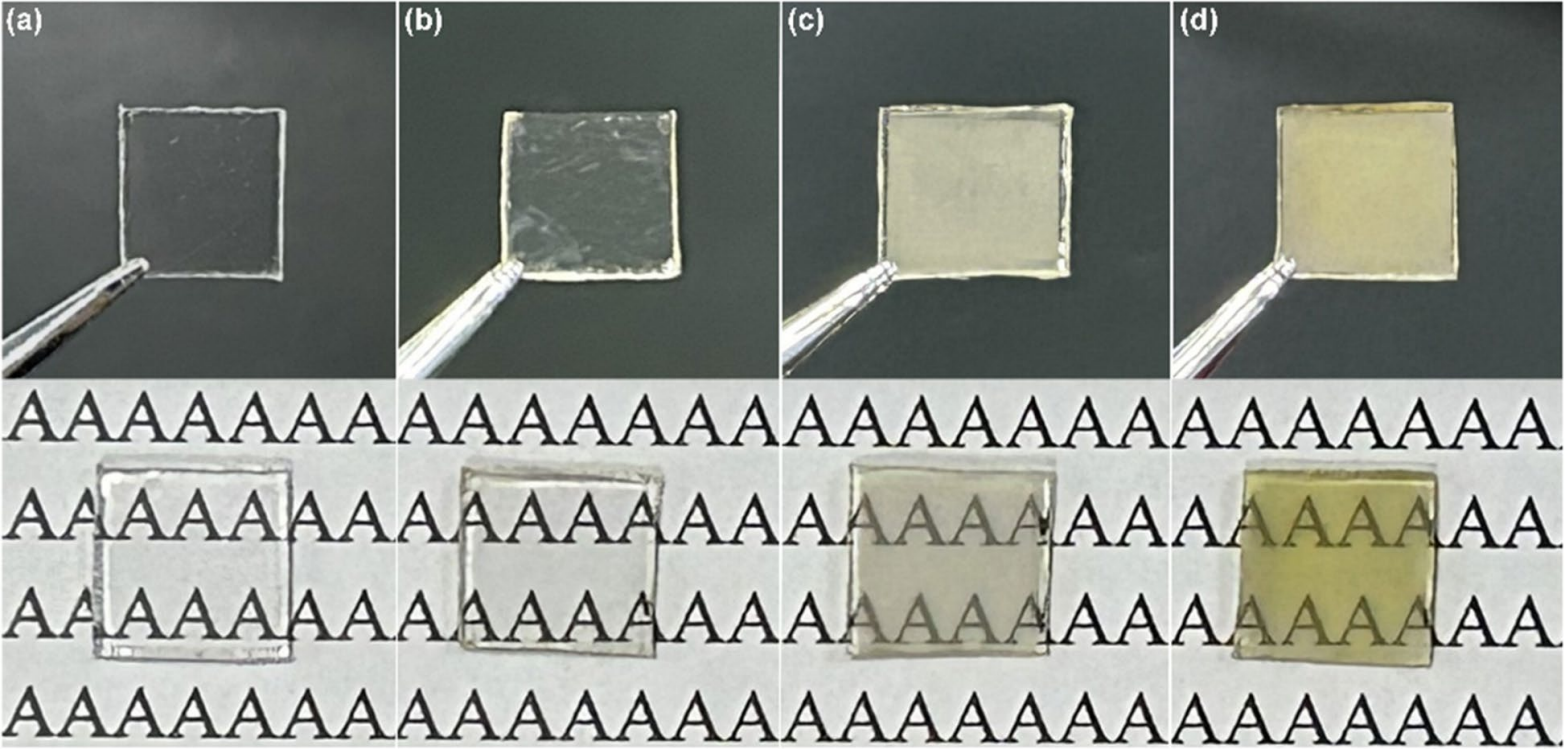
Photographs of (**a**) the control group, (**b**) the control/Ag group, (**c**) the laser group, (**d**) the laser/Ag group

Figure 4 shows photographs of the samples. The control group is colorless and transparent (Fig. 4a). The surface of the control/Ag group appears slightly blurred compared with the control group (Fig. 4b). In contrast, the laser-treated diaphragm exhibits a slight brownish-yellow coloration (Fig. 4c). After AgNP loading, the surface color further darkens, appearing brown (Fig. 4d). While these color changes are evident, all samples still maintain a level of transparency, although to a lesser degree compared to the control group, the underlying text remains visible.

### Coating durability

**Fig. 5:**
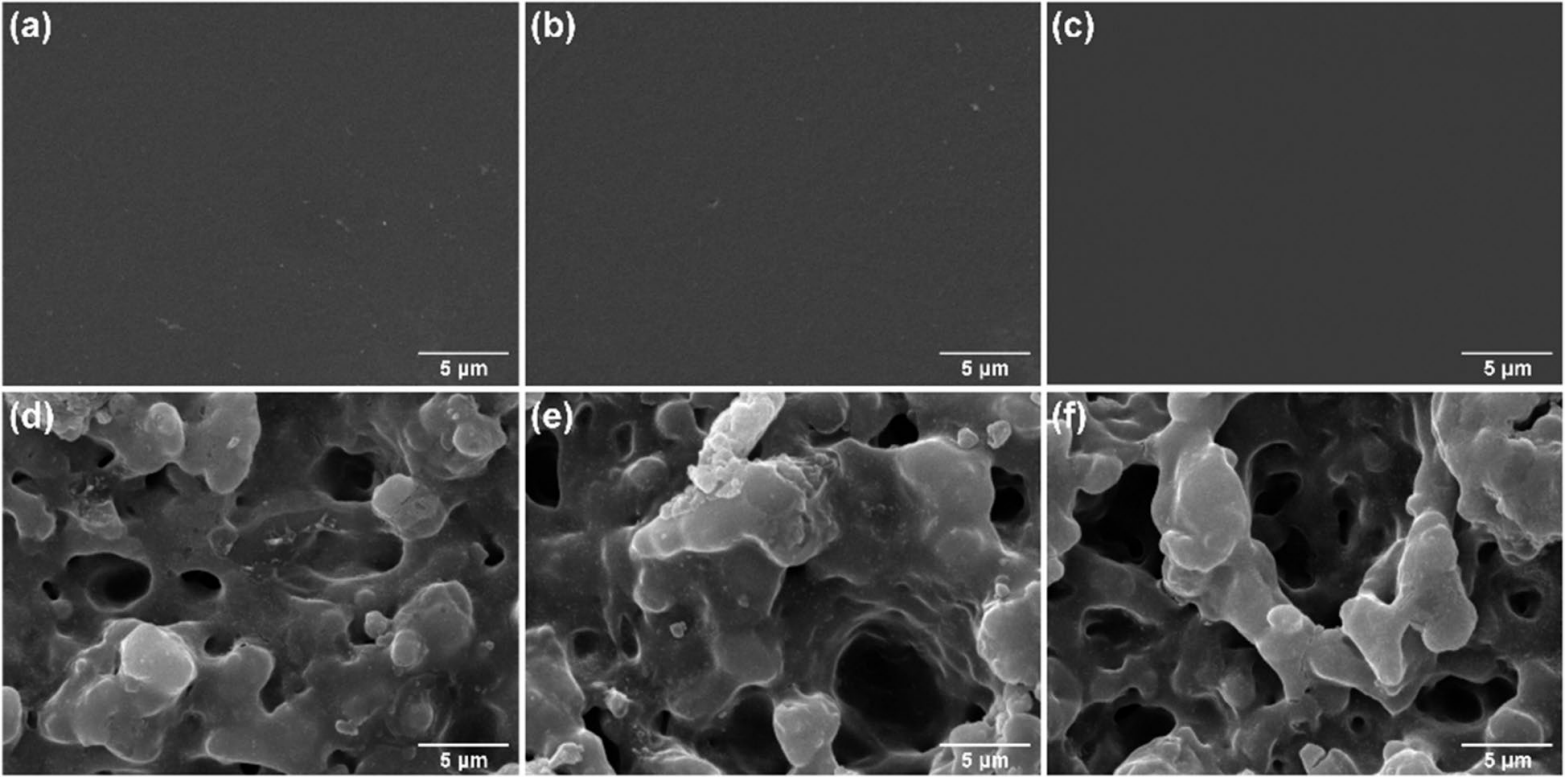
SEM images of (**a**) the control/Ag group, (**b**) the control/Ag group after immersion in artificial saliva, (**c**) the control/Ag group after brushing, (**d**) the laser/Ag group, (**e**) the laser/Ag group after immersion in artificial saliva, (**f**) the laser/Ag group after brushing

As shown in Fig. 5, the distribution of the AgNPs on the aligner diaphragm surface in the control/Ag group is sparse, indicating a low AgNP loading (Fig. 5a). In contrast, the laser/Ag group has a denser distribution of AgNPs, demonstrating that the laser-induced porous micro/nanostructures enhance AgNP loading (Fig. 5d). After immersion in artificial saliva for 7 days, the AgNPs in the control/Ag group were significantly reduced, with only a few particles remaining (Fig. 5b), whereas the laser/Ag group retained a relatively dense and well-distributed population of AgNPs (Fig. 5e), indicating superior coating stability under simulated oral conditions. Following brushing with a soft-bristle toothbrush, AgNPs in the control/Ag group were almost completely removed (Fig. 5c), while in the laser/Ag group only the superficially exposed AgNPs were lost, and those embedded within the micro/nanostructures remained largely intact (Fig. 5f). These results suggest that the laser-induced micro/nanostructures can effectively protect AgNPs, thereby enhancing the resistance of the coating to saliva erosion and mechanical wear.

### Antibacterial effects

**Fig. 6:**
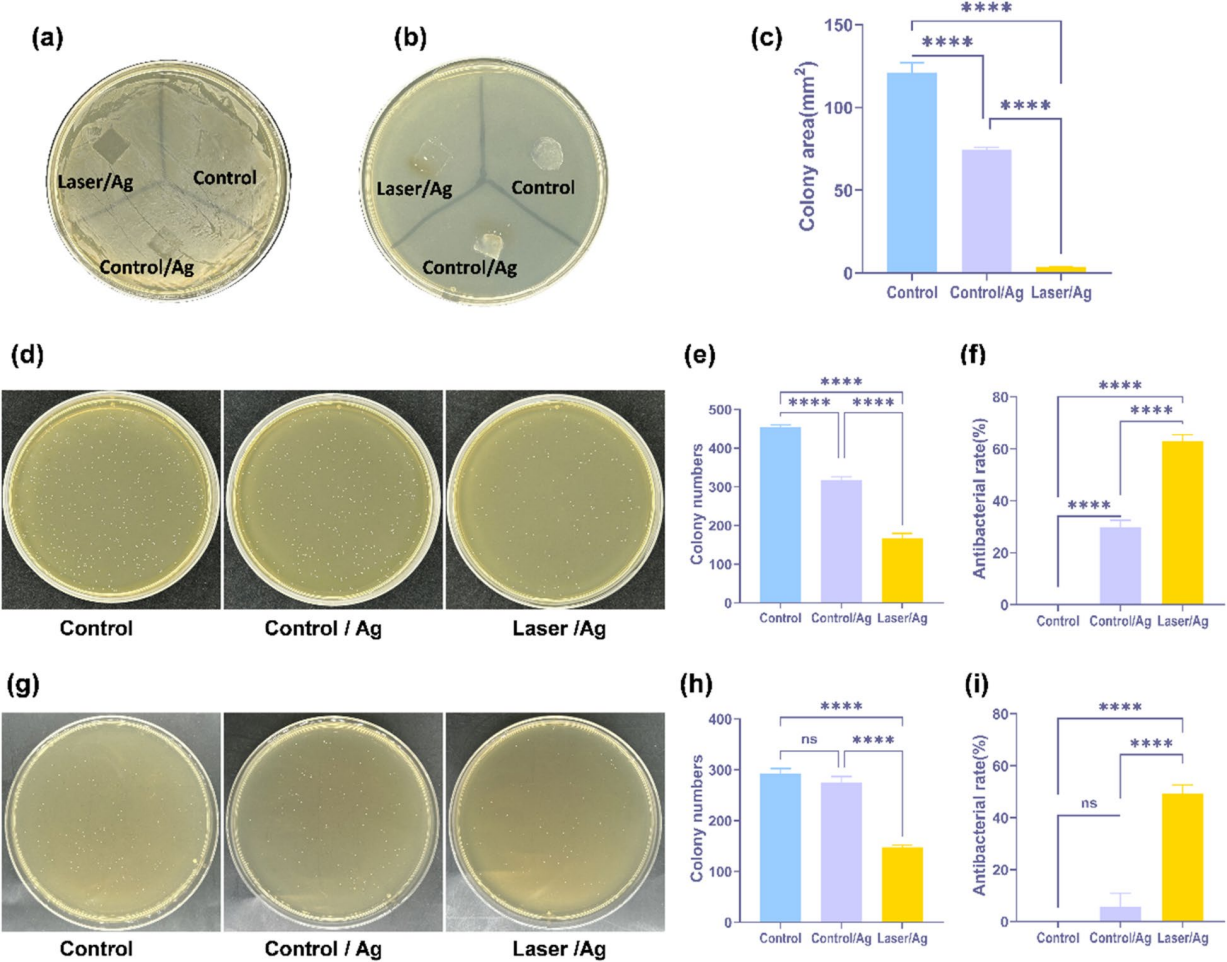
**a** Image of colony growth inhibition of *S. mutans* in three groups. **b** Images of growth area of three groups. **c** The size of colony growth area of corresponding area of three groups. **d** Colony growth results of *S. mutans* in three groups. **e** Colony numbers in three groups. **f** Antibacterial rate in three groups. **g** Colony growth results of *S. mutans* in three groups after immersion in saliva for 7 days. **h** Colony numbers in three groups after immersion in saliva for 7 days. **i** Antibacterial rate in three groups after immersion in saliva for 7 days. (****P < 0.0001)

The laser-treated diaphragm was immersed in an AgNP solution for 5 min to obtain diaphragms with superior bacteriostatic properties. *S. mutans* circle of inhibition and colony plate counting tests were used to characterize the antimicrobial activity of the samples. As shown in Fig. 6a, the white areas indicate the presence of *S. mutans* colonies, whereas the corresponding areas in the control group show almost unaffected colony growth. The control/Ag group exhibits a few transparent areas where most of the bacterial growth was present, whereas the laser/Ag group is completely transparent with almost no colony growth. Figure 6b shows an image of the *S. mutans* colony growth area in the contact area of the aligner diaphragm in the dish and the measured colony growth area. Statistical analysis using one-way ANOVA with Tukey's post-hoc test revealed significant differences in colony growth areas among the three groups (*F* = 879.7, *P* < 0.0001). The colony growth areas were 121.19 ± 5.85 mm^2^ (control), 74.65 ± 1.27 mm^2^ (control/Ag; adjusted *P* < 0.0001 vs control), and 3.5 ± 0.3 mm^2^ (laser/Ag; adjusted *P* < 0.0001 vs control and control/Ag) (Fig. 6c). As shown in Fig. 6d, S*. mutans* coated on untreated surfaces and cultured on BHI agar plates resulted in dense colonies, whereas fewer and sparser colonies were observed for the control/Ag and laser/Ag groups, with the laser/Ag group exhibiting the best antibacterial effect. Quantitative analysis of colony counts showed 453.33 ± 6.51 (control), 318 ± 7.55 (control/Ag; adjusted *P* < 0.0001 vs control), and 167 ± 12.53 (laser/Ag; adjusted *P* < 0.0001 vs control and control/Ag) (Fig. 6e), with one-way ANOVA confirming significant differences (*F* = 720.4, *P* < 0.0001).The inhibition rates were 0% (control), 29.71 ± 2.84% (control/Ag; adjusted *P* < 0.0001 vs control), and 62.89 ± 2.57% (laser/Ag; adjusted *P* < 0.0001 vs control and control/Ag) (Fig. 6f).

To evaluate the sustained antibacterial effect of AgNPs over time, the three groups were immersed in artificial saliva for 7 days before their antibacterial performance was assessed. As shown in Fig. 6g, S*. mutans* cultured on BHI agar plates formed dense colonies in the control and control/Ag groups, whereas markedly fewer and more sparsely distributed colonies were observed in the laser/Ag group, indicating sustained antibacterial activity. Quantitative analysis of colony counts revealed 291.67 ± 10.41 (control), 275.00 ± 11.79 (control/Ag; adjusted *P* < 0.0001 vs control), and 147.67 ± 4.51 (laser/Ag; adjusted *P* < 0.0001 vs control and control/Ag) (Fig. 6h), with one-way ANOVA confirming significant differences (*F* = 208.6, *P* < 0.0001). The inhibition rates were 0% (control), 5.63 ± 5.11% (control/Ag; adjusted *P* < 0.0001 vs control), and 49.29 ± 3.35% (laser/Ag; adjusted *P* < 0.0001 vs control and control/Ag) (Fig. 6i). Notably, the antibacterial activity of the control/Ag group decreased markedly after saliva immersion, whereas the laser/Ag group retained significant antibacterial efficacy despite showing a slight reduction, indicating that the laser-induced micro/nanostructures effectively retained AgNPs.

### Cytotoxicity

**Fig. 7:**
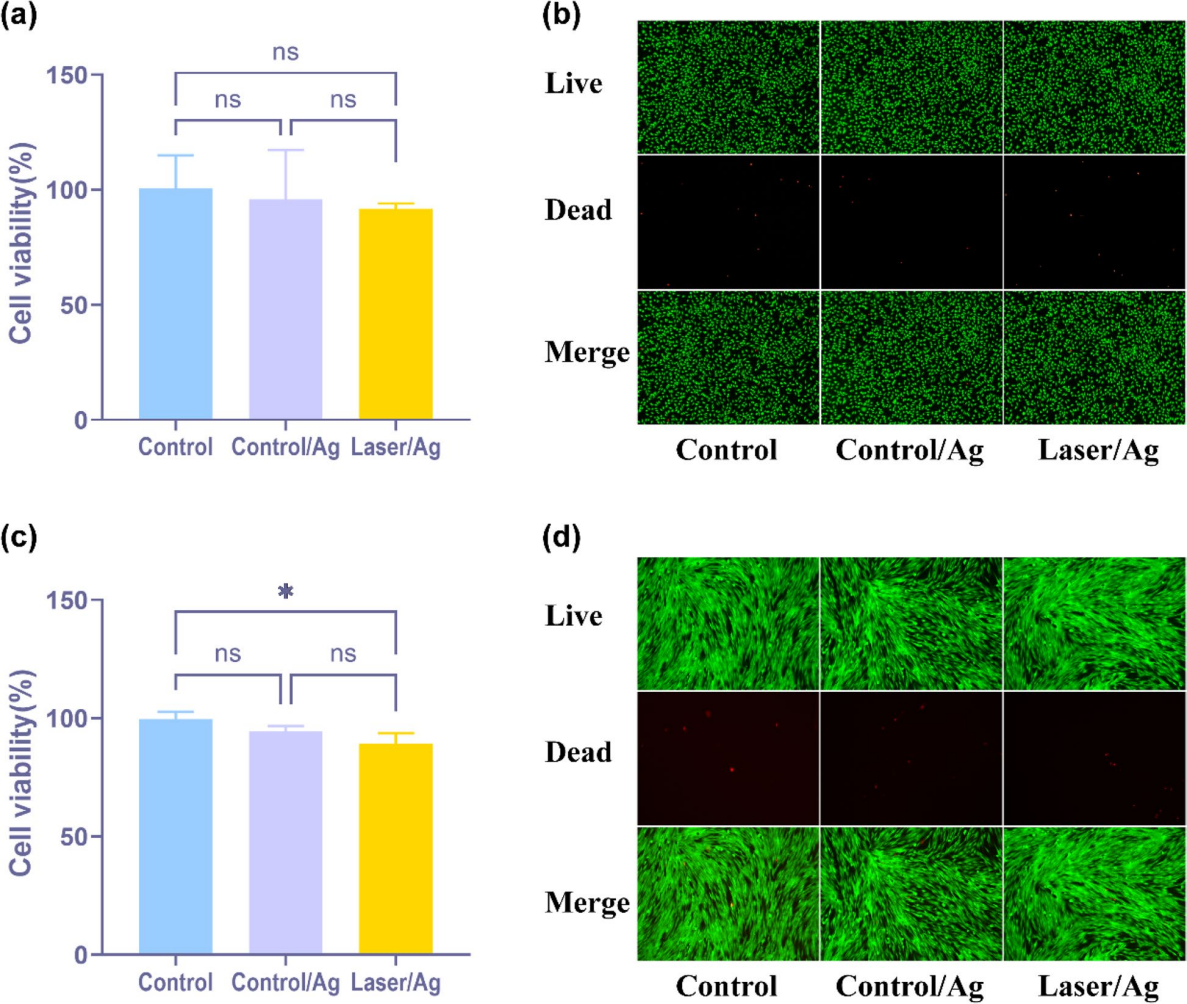
**a** Cell viability of L929 cells in the control, control/Ag, and laser/Ag groups (ns: *P* > 0.05). **b** Live/dead staining (Calcein AM/PI) of L929 cells in the control, control/Ag, and laser/Ag groups. **c** Cell viability of HGF cells in the control, control/Ag, and laser/Ag groups (ns: *P* > 0.05, *P < 0.05). (d) Live/dead staining (Calcein AM/PI) of HGF cells in the control, control/Ag, and laser/Ag groups

CCK-8 assay results (Fig. 7a and c) demonstrated comparable cell viability across all experimental groups. L-929 cell viability was comparable among the control (100.57 ± 14.33%), control/Ag (95.74 ± 21.54%), and laser/Ag (91.63 ± 2.46%) groups, with no significant differences detected by one-way ANOVA (*F* = 0.2667, *P* = 0.7745). In HGF cells, a statistically significant yet small-magnitude difference was observed (*F* = 7.061, *P* = 0.0265). Tukey's post-hoc test identified that this difference occurred specifically between the control (99.71 ± 3.04%) and laser/Ag (89.12 ± 4.57%) groups (adjusted *P* = 0.022). No significance was found between the control and control/Ag (94.31 ± 2.36%; adjusted *P* = 0.2152) or the control/Ag and laser/Ag groups (adjusted *P* = 0.2345). Notably, all tested samples maintained cell viability above 89%, well exceeding the 70% threshold mandated by international standards. These findings suggest that neither Ag incorporation nor laser treatment with Ag exerted cytotoxic effects on either cell line under the tested conditions. Live/dead staining with Calcein AM/PI (Fig. 7b and d) demonstrated robust cell viability across all experimental groups. Both L-929 and HGF cells exhibited predominantly green fluorescence, indicating a high proportion of viable cells with healthy spindle-shaped morphology characteristic of fibroblasts. Only sporadic red fluorescence (dead cells) was observed in the fields of view. There was no significant difference in the numbers of live and dead cells among the three groups. These findings further corroborate the CCK-8 assay results, confirming that the femtosecond laser-treated appliance diaphragm loaded with AgNPs exhibits good biocompatibility with oral cells. However, further clinical trials and long-term follow-up observations are required to confirm this conclusion.

## Discussion

The demineralization of tooth enamel during orthodontic treatment is an important issue. The loading of sufficient antimicrobial drugs on clear aligner surface provides many advantages, such as enhances antimicrobial effect, improves comfort, facilitates cleaning and maintenance. Therefore, this study proposed a novel method of applying femtosecond laser treatment to introduce micro/nanostructures on the surface of clear aligner to provide sufficient surface area for loading antimicrobial drugs.

The design of laser parameters has a huge impact on the behaviour of the surface structure of the material. Femtosecond laser light sources come in a wide range of wavelengths, commonly including infrared, green, and ultraviolet, and there are differences in the mechanisms by which lasers of different wavelengths interact with materials. The laser processing parameters and material properties critically influence the ablation of organic materials by short-pulse lasers, and the energy per unit area is the key parameter controlling the structure and morphology [23, 24]. Furthermore, parameters such as the laser power, repetition rate, pulse width, scanning speed, and processing spacing affect the accumulation of laser energy on the material surface, thus affecting the resulting micro/nanostructures. In a previous study, a femtosecond laser was used to construct a stepped structure on the surface of polydimethylsiloxane by multilayer etching, and a close correlation between the laser etching depth and energy density was observed [25]. A study of the effect of femtosecond laser processing parameters on the morphology of polytetrafluoroethylene showed that a low laser energy density during single-pulse ablation resulted in unclear pit edges and irregularly shaped features [26]. In addition to surface morphology regulation, processing time is an important factor in evaluating the practical applicability of femtosecond laser treatment. For a PETG diaphragm with a diameter of 125 mm, as used for fabricating a single dental arch aligner, the processing time is approximately 5 min under the current conditions (scanning speed of 2000 mm/s and hatch spacing of 20 µm) when full-area scanning is applied. Although this processing time is acceptable for laboratory-scale preparation and proof-of-concept studies, it may limit the efficiency of large-scale personalized production of clear aligners. Therefore, future work should focus on optimizing processing strategies, such as reducing unnecessary scanning paths and applying selective surface modification to clinically relevant regions that are more prone to bacterial accumulation. These approaches may help reduce processing time while maintaining the desired surface functionality, thereby improving production efficiency and supporting the potential clinical application of this technique.

Materials can be laser-processed to form superhydrophobic surfaces, effectively preventing bacterial adhesion. However, this outcome is also highly dependent on the material’s inherent properties. Zhou et al. utilized a femtosecond laser to fabricate biomimetic microstructures on polytetrafluoroethylene polymer surfaces, successfully obtaining superhydrophobic and superoleophobic surfaces with antifouling and self-cleaning characteristics [27]. Clear aligner materials are mostly thermoplastic polypropylene and copolyester polymers [28]. The aligner diaphragm used in this study was composed of a PETG-based thermoplastic. Kwok et al. identified the FTIR absorption peaks of C–H bonds, carbonyl groups, and C–O bonds at 2900, 1700, and 1250 cm-1, respectively [29], and our results are consistent with these assignments. The FTIR results show that the chemical and molecular composition of the aligner diaphragm material did not change significantly after laser treatment. Femtosecond laser treatment is a complex nonlinear process affecting the surface of polymer aligner diaphragms. In the area near the laser focal point, the surface material is removed when the laser energy is small. When the laser energy is large, the laser beam interacts with the material to form plasma expansion, resulting in high local temperatures and pressures that eject material from the surface, which falls back to the surface and forms multi-level micro/nanostructures upon solidification. Given that the aligner diaphragm is inherently a hydrophilic material, preparing a superhydrophobic surface presents significant challenges. This could potentially be achieved in future research by further exploring laser parameters and designing secondary surface structures. Additionally, aesthetics is a very important advantage of invisible aligners, and various antimicrobial strategies must be considered if they are to be used clinically in terms of their effect on staining. Worreth et al. used polyethyleneimine as a cross-linking agent to construct a curcumin-chitosan antimicrobial coating on the clear aligner surface [30]. Qin et al. developed a multifunctional coating TA-Cu MPNs@ZDS@CaP with antimicrobial and remineralization effects [31]. These coatings have the unfavorable effect of staining the aligner. In laser processing, precise parameter adjustment is necessary to control laser energy, as too much energy may cause discoloration of the substrate.

Surface micro/nanostructure modification can change the hydrophilic and hydrophobic properties of the material surface [32]. Wenzel's theory describes the relationship between surface roughness and wetting behavior. When the surface becomes rough, as in the case of the laser textured surfaces prepared in this study, the droplets can interact with a larger surface area owing to the rough protrusions and micropores [33]. For hydrophilic materials, roughness enhances the interaction of water with the solid surfaces, making droplets more diffuse and lowering the WCA compared to a smooth surface. In this study, the WCA of the appliance diaphragm decreased from 86.5° to 38.7° after femtosecond laser treatment, i.e., the surface hydrophilicity increased significantly, which is attributed to the development of the multi-level micro/nanostructure that increases the surface area of the diaphragm.

The force applied to an invisible orthodontic by moving teeth mainly comes from the resilience force after the deformation of the hot compression film material. The ideal orthodontic tooth movement involves a continuous light force, which requires the material to have high strength and appropriate stiffness. The flexural modulus of elasticity is an important characteristic of a material’s mechanical properties and is used as an index to measure the difficulty of elastic deformation. A material with a high flexural modulus of elasticity has low elastic deformation and high stiffness. Many studies have shown that the type and thickness of the diaphragm have the greatest influence on its elastic modulus. Maki et al. studied the mechanical properties, water absorption, and light transmittance of eight dental materials and found that the properties of the materials were significantly affected by the environment and their structure [34]. Kohda et al. confirmed that thicker appliance materials (0.75 or 0.8 mm) always produced more orthodontic force than thinner ones (0.4 or 0.5 mm) [35]. In this study, the femtosecond laser treatment of the aligner diaphragm achieved nanoscale modification under cold-working conditions, which had a small effect on the thickness and structure of the material. Therefore, the mechanical properties of the aligner diaphragm were not significantly affected by the surface treatment, thereby satisfying the basic force application requirements of the aligner.

The saliva absorption rate of the aligner diaphragm can affect the chemical bonding between the molecules of the material, which enhances water absorption and changes its physical and chemical properties, thus accelerating its aging [36]. In this study, although laser processing increases the hydrophilicity of the diaphragm and the contact area of the diaphragm, it is a cold process, and precise cuts do not change the chemical structure of the diaphragm surface. Therefore, laser processing does not increase the saliva uptake of the orthodontic diaphragm or affect its functionality. Because the appliance needs to be worn for a long time, the water absorption and resulting expansion of the diaphragm may change the size and fit of the aligner, which affects its mechanical performance [37]. Therefore, the water absorption of the invisible appliance material should be as low as possible. The water absorption of a material is affected by various factors, such as the type of material, internal crystallinity, and structural hierarchy. Although the hydrophilicity of the surface of the appliance diaphragm was significantly higher after laser treatment, the changes to the diaphragm are limited to the outer surface of the material, so no significant difference in the saliva absorption rate was observed between the two sample groups.

The loading of drugs on micro/nanostructures has a wide range of applications in the oral cavity, as demonstrated by the porous structure of hydrogels and electrospun materials [38, 39]. In this study, an innovative micro/nanostructured drug delivery system prepared using femtosecond laser treatment is proposed, which avoids the use of raw materials for the drug delivery system and effectively promotes the attachment and storage of AgNPs by directly changing the morphology and wettability of the material surface. The SEM results show that the laser group can carry more AgNPs than the control group because the porous micro/nanostructure increases the surface area, which significantly improves the drug-loading capacity of the diaphragm. Invisible aligners are removable appliances, and their surfaces are subjected to repeated friction during use. The 3D microstructures loaded with AgNPs showed that the coatings were less affected by removal and wear compared to PETG in the control group, which implies that the AgNPs were more likely to be retained. Most of the existing antimicrobial strategies are to prepare coatings on 2D aligner diaphragm and the coating preparation time is long, this study provides a new idea for the preparation of antimicrobial coatings for invisible aligners. In addition, the hydrophilicity of the diaphragm surface increases after laser treatment, which is beneficial for loading the AgNPs. Furthermore, the femtosecond laser treatment is simpler and faster than the processes presented in previous studies.

In this study, the broad size distribution of AgNPs synthesized using tea polyphenols can be attributed to multiple factors. Tea polyphenols represent a complex mixture of various phenolic compounds, including catechins and epicatechins, which exhibit significantly different reducing activities. Highly active components such as gallate esters rapidly reduce silver ions to form small nuclei, while less active components continue to participate in the later stages of reaction, resulting in heterogeneous nucleation and growth rates [40, 41]. Concurrently, minor fluctuations in reaction conditions—including temperature, pH, and reactant concentrations—alter the kinetics of silver ion reduction and nanocrystal growth. Specifically, localized temperature variations affect reaction rates, pH changes modulate polyphenol activity and silver ion speciation, and concentration gradients lead to imbalanced nucleation and growth processes [42]. Ni et al. found that the reaction time, pH, and concentration of plant extracts affected the particle size of the final AgNPs [43]. Quiroz et al. found that small spherical particles can be obtained at alkaline pH; however, excessive pH increases the diameter of AgNPs, resulting in reduced particle aggregation and synthesis rates [44]. These interconnected factors collectively contribute to the observed polydispersity in AgNP sizes. In addition, the particle size distribution is closely associated with the relative rates of nucleation and subsequent particle growth. Higher silver ion concentrations or prolonged reaction times may increase the extent of nucleation, but they also favor continued crystal growth and particle aggregation, ultimately leading to larger and more polydisperse nanoparticles [45, 46]. In contrast, increasing the concentration of tea polyphenols may accelerate the initial reduction process while enhancing surface capping and stabilization, thereby promoting the formation of smaller and more uniform nanoparticles [47, 48]. Therefore, improving particle size control requires a better balance between nucleation and growth during synthesis. This may be achieved by optimizing the AgNO₃-to-tea-polyphenol ratio, reaction pH, temperature, and reaction time. In addition, post-synthesis treatments, such as ultrasonic dispersion, gradient centrifugation, or membrane filtration, may further remove large particles and aggregates, thereby narrowing the size distribution and improving nanoparticle uniformity.

AgNPs are an antibiotic-free antimicrobial agent and their antimicrobial activity involves the following mechanism. The Ag + released from the AgNPs adheres to the cell walls and membranes owing to electrostatic attraction and affinity for sulfhydryl groups, which enhances the permeability of the cell membrane, leading to the destruction of the bacterial envelope [49]. After Ag + enters bacterial cells, it binds to thiophene or guanine in DNA, which blocks bacterial DNA replication, leading to cell mutation or apoptosis [50]. In addition, AgNPs and their released Ag + inactivate respiratory chain dehydrogenase and generate excessive reactive oxygen species, leading to a decrease in ATP production, disrupting its life activities, and leading to bacterial death [51]. These synergistic effects are the source of the outstanding antimicrobial properties of AgNPs. In this study, owing to the improved drug-loading performance after femtosecond laser treatment, the antimicrobial effect of the laser/Ag surfaces increased by approximately 30% compared to the control/Ag surfaces. This shows that the laser/Ag treatment of the diaphragms resulted in excellent antimicrobial properties and effectively killed the bacteria in contact with the surfaces.

Enamel demineralization is primarily driven by the acidogenic activity of dental biofilms, which lowers the local pH to a critical threshold of approximately 5.5, below which mineral dissolution occurs and promotes caries development [52, 53]. Previous studies indicate that salivary *S. mutans* levels of ≥ 10^5^ CFU/mL are indicative of high caries risk [54, 55]. However, cariogenicity is not solely determined by bacterial counts but also depends on biofilm architecture and the formation of an acidic microenvironment [56, 57]. Therefore, no definitive antibacterial threshold has yet been established for the level of bacterial reduction required to completely prevent white spot lesions or caries. In this study, a high bacterial load (10⁸ CFU/mL) was employed to simulate a stringent cariogenic challenge. Under these conditions, the laser/Ag group still achieved an antibacterial rate of 62.89%, demonstrating a substantial inhibitory effect on *S. mutans*. This finding suggests that the coating can effectively reduce the acidogenic potential of biofilms and thereby lower the risk of enamel demineralization. Notably, the antibacterial performance could be further enhanced by synthesizing smaller-sized AgNPs or by loading other highly effective antimicrobial agents. In conclusion, laser-treated diaphragms loaded with silver nanoparticles exhibit promising antibacterial properties and potential for clinical application.

The antimicrobial strategies for clear aligners primarily include the fabrication of antibacterial coatings and antibacterial composite materials, as summarized in Table 1 at the end of the article. Surface coating technology is an important approach to enhance the antimicrobial properties of aligners. Antimicrobial coating materials can generally be classified into two categories: inorganic compounds and organic substances [5, 58]. Significant differences exist in the preparation methods, bonding mechanisms, and performance characteristics of various antimicrobial coatings [59, 60]. When considering preparation efficiency and antimicrobial durability, chemically bonded strategies—achieved through metal–ligand coordination or covalent grafting—provide stable coating anchorage [61, 62]. Although these methods require prolonged preparation cycles, their abrasion resistance and antimicrobial persistence markedly surpass those of physical adsorption systems [63]. Conversely, the physical adsorption-dominated TiO₂-Cu aerosol coating can rapidly form a film by high-speed deposition [64]. This limitation renders them prone to nanoparticle detachment under mechanical friction or salivary flow, ultimately diminishing antimicrobial efficacy. The micro/nanostructure-assisted physical loading of silver nanoparticles (AgNPs) developed in this study demonstrates distinct advantages over existing technologies: (1) high efficiency, achieving robust antimicrobial effects with only 5 min of immersion—a drastic improvement over traditional chemical bonding processes requiring 12–24 h; and (2) repeatability, as the micro/nanotextured surface facilitates reagent-free reloading of AgNPs without reliance on complex chemical bonds or harsh reaction conditions, offering practical clinical benefits. Given that patients typically wear aligners for 20–22 h daily, even partial attenuation of antibacterial efficacy after 24 h allows sufficient time for convenient AgNPs replenishment, ensuring uninterrupted antimicrobial protection throughout treatment. Furthermore, integrating antimicrobial materials into aligner substrates represents another viable strategy. While composite modifications (e.g., PETG/BaTiO₃NPs) leverage inherent material properties for sustained protection [65], technical challenges persist, particularly in achieving uniform nanoparticle dispersion within the substrate. Inhomogeneous distribution may compromise mechanical performance and create localized variations in antimicrobial activity, adversely affecting device quality and clinical outcomes.

**Table 1:** Comparison of antimicrobial strategies of clear aligners

DOI	Antibacterial materials	Aligner material	Preparation methods	Connection method	preparation time
10.3389/fbioe.2024.1418493	TA-Cu MPNs @ZDS@CaP	Aligner diaphragms	Soaking	Chemical bonding	12 h
10.1016/j.cej.2024.153851	TiO_2_-Cu	PETG	Aerosol deposition	Physical adsorption	_______
10.1111/ocr.12899	ZnO, MgO, ZnO + MgO	PETG	Aminosilane treatment, and then soaking	Chemical bonding	24 h
10.1021/acsbiomaterials.9b01647	QAGNCs	Invisalign aligner	Oxygen plasma treatment, and then soaking	Electrostatic self-assembly	12 h
10.1021/acsomega.0c01532	AuDAPT	Invisalign aligner	Oxygen plasma treatment, and then soaking	Electrostatic self-assembly	12 h
10.1016/j.ajodo.2021.11.020	ZnO	Invisalign aligner	Magnetron sputtering	Physical adsorption	______
10.1002/marc.202400234	G-co-S and G-g-S	PETG	Soaking	Chemical bonding	6 h
10.1021/acsami.8b04433	CMC/CHI	PETG	LbL assembly and cross-linking	Chemical bonding	70 min
10.1016/j.apsusc.2021.152085	PSQ	Clear Overlay Appliance	Oxygen plasma treatment, and then soaking	Chemical bonding	1 h
10.1016/j.colsurfb.2022.112696	hydrogel matrix and octapeptides	Erkodur discs	Soaking	Chemical bonding	1 h
10.1038/s41598-023–39320-1	Essential oils	Cellulose-based multilayered thermoformable material	Soaking	Physical adsorption	1 h
10.1007/s00784-023-05216-7	Fluoride	Angelalign aligner	_____	_____	_____
10.3390/s23115336	BaTiO_3_NPs	PETG	Solution blending	_____	_____
10.3390/nano13192649	Chitosan nanoparticles	Clear resin (CR)	Ultrasonic dispersion	_____	_____

However, further studies are required to improve upon the presented results. First, the femtosecond laser parameters need to be further explored to prepare multiscale micro/nanostructures on clear aligner surface and reduce color changes. Moreover, the long-term durability of the coating under mechanical stress remains uninvestigated, raising concerns about its clinical longevity and performance over extended treatment periods. These limitations highlight the need for future research to enhance the coating’s mechanical resilience, evaluate staining risks under clinically relevant conditions, and develop strategies to mitigate these challenges. Therefore, addressing these gaps will not only improve the translational potential of this technology but also advance the development of durable and aesthetically favorable antimicrobial orthodontic biomaterials. A future research direction for our group is the development of bionic micro/nanostructure on the surface of invisible aligners by adjusting the femtosecond laser parameters to minimize the adhesion and aggregation of bacteria. This is clinically important for minimizing the use of antimicrobial drugs and ensuring long-term antimicrobial stability.

## Conclusion

This study demonstrated the combination of femtosecond laser treatment and AgNP antimicrobial coating for invisible aligners. Porous micro/nanostructures were successfully formed on the surface of invisible aligner diaphragms using femtosecond laser treatment, resulting in enhanced surface area and hydrophilicity for the effective loading and storage of AgNPs. Aligner diaphragms loaded with AgNPs effectively killed *Streptococcus mutans*, inhibited biofilm formation, and exhibited excellent biocompatibility. Notably, the modified surface structure does not affect the chemical composition, saliva absorption, or mechanical properties of the material. This innovative drug delivery platform demonstrates versatile applicability with broad material compatibility for hydrophilic nano-antimicrobials, establishing a clinically translatable framework for orthodontic infection control.

## Supplementary Information

Supplementary Material 1.

## Data Availability

Full data can be made available upon reasonable request to the corresponding author.

## Abbreviations

AgNPs: Silver nanoparticles
HGF: Human gingival fibroblast
L-929: Mouse fibroblast
*S. mutans*: *Streptococcus mutans*
PBS: Phosphate-buffered saline
SEM: Scanning electron microscopy
EDS: Energy-dispersive X-ray spectroscopy
XPS: X-ray photoelectron spectroscopy
FTIR: Fourier transform infrared spectroscopy
WCA: Water contact angle
UV–vis: UV–visible spectrophotometer
FE-SEM: Field emission scanning electron microscope
CFU: Colony-forming unit
BHI: Brain heart infusion
CCK-8: Cell counting kit-8
OD: Optical density
SD: Standard deviation
ANOVA: One-way analysis of variance
PETG: Polyethylene terephthalate glycol
ATP: Adenosine triphosphate
TiO₂: Titanium dioxide
Cu: Copper
ZnO: Zinc oxide
MgO: Magnesium oxide
CMC: Carboxymethyl cellulose
CHI: Chitosan
PSQ: Polysiloxane
BaTiO₃NPs: Barium titanate nanoparticles

## References

[1] Paim J, Souza LFD, Fialho T, Borba DBM, Freitas KMS, Cotrin P, et al. Assessment of patients’ knowledge and preferences for the use of orthodontic aligners. J Orthod. 2024;51:14653125241229456. 10.1177/14653125241229456. 10.1177/14653125241229456 38323415

[2] Bichu YM, Alwafi A, Liu X, Andrews J, Ludwig B, Bichu AY, et al. Advances in orthodontic clear aligner materials. Bioactive Mater. 2023;22:384–403. 10.1016/j.bioactmat.2022.10.006. 10.1016/j.bioactmat.2022.10.006 PMC9588987 36311049

[3] Rouzi M, Zhang X, Jiang Q, Long H, Lai W, Li X. Impact of clear aligners on oral health and oral microbiome during orthodontic treatment. Int Dental J. 2023;73:603–11. 10.1016/j.identj.2023.03.012. 10.1016/j.identj.2023.03.012 PMC10509397 37105789

[4] Song Z, Fang S, Guo T, Wen Y, Liu Q, Jin Z. Microbiome and metabolome associated with white spot lesions in patients treated with clear aligners. Front Cell Infect Microbiol. 2023;13:1119616. 10.3389/fcimb.2023.1119616. 37082715 10.3389/fcimb.2023.1119616 PMC10111054

[5] Wang N, Yu J, Yan J, Hua F. Recent advances in antibacterial coatings for orthodontic appliances. Front Bioeng Biotechnol. 2023;11:1093926. 10.3389/fbioe.2023.1093926. 36815889 10.3389/fbioe.2023.1093926 PMC9931068

[6] Hamouda RA, Makharita RR, Qarabai FAK, Shahabuddin FS, Saddiq AA, Bahammam LA, et al. Antibacterial activities of ag/cellulose nanocomposites derived from marine environment algae against bacterial tooth decay. Microorganisms. 2023;12:1. 10.3390/microorganisms12010001. 38276170 10.3390/microorganisms12010001 PMC10820646

[7] Odatsu T, Kuroshima S, Sato M, Takase K, Valanezhad A, Naito M, et al. Antibacterial properties of nano-ag coating on healing abutment: an in vitro and clinical study. Antibiotics (Basel, Switzerland). 2020;9:347. 10.3390/antibiotics9060347. 32575552 10.3390/antibiotics9060347 PMC7345643

[8] Xie Y, Zhang M, Zhang W, Liu X, Zheng W, Jiang X. Gold nanoclusters-coated orthodontic devices can inhibit the formation of streptococcus mutans biofilm. ACS Biomater Sci Eng. 2020;6:1239–46. 10.1021/acsbiomaterials.9b01647. 33464842 10.1021/acsbiomaterials.9b01647

[9] Vas NV, Jain RK, Ramachandran SK. Gingerol and chitosan-based coating of thermoformed orthodontic aligners: characterization, assessment of anti-microbial activity, and scratch resistance: an in vitro study. Cureus. 2023;15:e42933. 10.7759/cureus.42933. 37674946 10.7759/cureus.42933 PMC10477816

[10] Paradowska-Stolarz A, Mikulewicz M, Laskowska J, Karolewicz B, Owczarek A. The importance of chitosan coatings in dentistry. Marine drugs. 2023;21:613. 10.3390/md21120613. 38132934 10.3390/md21120613 PMC10744558

[11] K. Schwibbert, A.M. Richter, J. Krueger, and J. Bonse, Laser-Textured Surfaces: A Way to Control Biofilm Formation? Laser & Photonics Reviews 18 (2024).10.1002/lpor.202300753.

[12] Zhou Z, Ma B, Zhang X, Tang L, Lin X, Hu C, et al. Enhanced corrosion resistance and self-cleaning properties of superhydrophobic nickel coating fabricated by one-step electrodeposition. Ceramics Int. 2023;49:13109–18. 10.1016/j.ceramint.2022.12.188.

[13] Unno N, Mäkelä T. Thermal nanoimprint lithography-a review of the process, mold fabrication, and material. Nanomaterials (Basel, Switzerland). 2023;13:2031. 10.3390/nano13142031. 37513042 10.3390/nano13142031 PMC10385880

[14] Athira ET, Satija J. Plasmonic nanoparticle etching-based optical sensors: current status and future prospects. Analyst. 2023;148:6188–200. 10.1039/d3an01244a. 37916263 10.1039/d3an01244a

[15] Bhattacharyya A, Tiwari V, Karmakar T. Electrostatic-driven self-assembly of janus-like monolayer-protected metal nanoclusters. J Phys Chemist Lett. 2024;15:687–92. 10.1021/acs.jpclett.3c03508. 10.1021/acs.jpclett.3c03508 38206834

[16] Alonzo SMM, Bentley J, Desai S, Bastakoti BP. Hydrothermal synthesis of hierarchical microstructure tungsten oxide/carbon nanocomposite for supercapacitor application. Sci Rep. 2023;13:21732. 10.1038/s41598-023-48958-w. 38066064 10.1038/s41598-023-48958-w PMC10709354

[17] Zhang W, Li S, Wei D, Zheng Z, Han Z, Liu Y. Fluorine-free, robust and self-healing superhydrophobic surfaces with anticorrosion and antibacterial performances. J Mat Sci Technol. 2024;186:231–43. 10.1016/j.jmst.2023.10.059.

[18] Ma Y, Jiang L, Hu J, Liu H, Wang S, Zuo P, et al. Multifunctional 3D micro-nanostructures fabricated through temporally shaped femtosecond laser processing for preventing thrombosis and bacterial infection. ACS Appl Mater Interfaces. 2020;12:17155–66. 10.1021/acsami.9b20766. 31990516 10.1021/acsami.9b20766

[19] Yin K, Wang L, Deng Q, Huang Q, Jiang J, Li G, et al. Femtosecond laser thermal accumulation-triggered micro-/nanostructures with patternable and controllable wettability towards liquid manipulating. Nanomicro Lett. 2022;14:97. 10.1007/s40820-022-00840-6. 35394233 10.1007/s40820-022-00840-6 PMC8993985

[20] D Zhu, P Zuo, F Li, H Tian, T Liu, L Hu, H Huang, J Liu, X Qian, Fabrication and applications of surface micro/nanostructures by femtosecond laser. Colloid and Interface Science Communications. 2024;59. 10.1016/j.colcom.2024.100770.

[21] R.I. Rodríguez-Beltrán, J. Prada-Rodrigo, A. Crespo, T.A. Ezquerra, P. Moreno, and E. Rebollar, Physicochemical Modifications on Thin Films of Poly(Ethylene Terephthalate) and Its Nanocomposite with Expanded Graphite Nanostructured by Ultraviolet and Infrared Femtosecond Laser Irradiation. Polymers. 2022;14.10.3390/polym14235243. 10.3390/polym14235243 PMC9737047 36501637

[22] E Rebollar, JR Vazquez de Aldana, JA Perez-Hernandez, TA Ezquerra, P Moreno, M Castillejo. Ultraviolet and infrared femtosecond laser induced periodic surface structures on thin polymer films. Applied Physics Letters. 2012;100.10.1063/1.3679103.

[23] L Yang, J Wei, Z Ma, P Song, J Ma, Y Zhao, Z Huang, M Zhang, F Yang, X Wang. The Fabrication of Micro/Nano Structures by Laser Machining. Nanomaterials (Basel, Switzerland). 2019;9.10.3390/nano9121789. 10.3390/nano9121789 PMC6956144 31888222

[24] J. Bonse, Quo Vadis LIPSS?-Recent and Future Trends on Laser-Induced Periodic Surface Structures. Nanomaterials (Basel, Switzerland) 10 (2020).10.3390/nano10101950. 10.3390/nano10101950 PMC7601024 33007873

[25] Yong J, Yang Q, Chen F, Zhang D, Du G, Bian H, et al. Superhydrophobic PDMS surfaces with three-dimensional (3D) pattern-dependent controllable adhesion. Appl Surface Sci. 2014;288:579–83. 10.1016/j.apsusc.2013.10.076.

[26] Wang ZB, Hong MH, Lu YF, Wu DJ, Lan B, Chong TC. Femtosecond laser ablation of polytetrafluoroethylene (Teflon) in ambient air. J Appl Phys. 2003;93:6375–80. 10.1063/1.1568154.

[27] Zhou S, Hu Y, Huang Y, Xu H, Wu D, Wu D, et al. Preparation of polytetrafluoroethylene superhydrophobic materials by femtosecond laser processing technology. Polymers. 2023;16:43. 10.3390/polym16010043. 38201708 10.3390/polym16010043 PMC10780796

[28] Daniele V, Macera L, Taglieri G, Spera L, Marzo G, Quinzi V. Color stability, chemico-physical and optical features of the most common PETG and PU based orthodontic aligners for clear aligner therapy. Polymers. 2021;14:14. 10.3390/polym14010014. 35012036 10.3390/polym14010014 PMC8747426

[29] MH Kwok, B Porto, S Mohebi, L Zhu, M Hans. Physical and chemical properties of five different clear thermoplastic materials. Journal of Applied Polymer Science 139 (2022).10.1002/app.51957.

[30] Worreth S, Bieger V, Rohr N, Astasov-Frauenhoffer M, Töpper T, Osmani B, et al. Cinnamaldehyde as antimicrobial in cellulose-based dental appliances. J Appl Microbiol. 2022;132:1018–24. 10.1111/jam.15283. 34480822 10.1111/jam.15283 PMC9292871

[31] Qin Q, Yuan W, Zhang J, Gao Y, Yu Y. A pH-sensitive, renewable invisible orthodontic aligners coating manipulates antibacterial and in situ remineralization functions to combat enamel demineralization. Front Bioeng Biotechnol. 2024;12:1418493. 10.3389/fbioe.2024.1418493. 39108594 10.3389/fbioe.2024.1418493 PMC11301642

[32] Tang X, Tian Y, Tian X, Li W, Han X, Kong T, et al. Design of multi-scale textured surfaces for unconventional liquid harnessing. Mater Today. 2021;43:62–83.

[33] A.O. Ijaola, E.A. Bamidele, C.J. Akisin, I.T. Bello, A.T. Oyatobo, A. Abdulkareem, P.K. Farayibi, and E. Asmatulu, Wettability Transition for Laser Textured Surfaces: A Comprehensive Review. Surfaces and Interfaces 21 (2020).10.1016/j.surfin.2020.100802.

[34] M.K.J.O.W. Maki, The mechanical properties of dental thermoplastic materials in a simulated intraoral environment. 2006

[35] Kohda N, Iijima M, Muguruma T, Brantley WA, Ahluwalia KS, Mizoguchi I. Effects of mechanical properties of thermoplastic materials on the initial force of thermoplastic appliances. Angle Orthod. 2013;83:476–83. 10.2319/052512-432.1. 23035832 10.2319/052512-432.1 PMC8763061

[36] Ihssen BA, Willmann JH, Nimer A, Drescher D. Effect of in vitro aging by water immersion and thermocycling on the mechanical properties of PETG aligner material. J Orofacial Orthop Fortschritte der Kieferorthopadie. 2019;80:292–303. 10.1007/s00056-019-00192-8. 10.1007/s00056-019-00192-8 31578595

[37] Zhang N, Bai Y, Ding X, Zhang Y. Preparation and characterization of thermoplastic materials for invisible orthodontics. Dental Mater J. 2011;30:954–9. 10.4012/dmj.2011-120. 10.4012/dmj.2011-120 22123023

[38] Yang Y, Zhang R, Liang Z, Guo J, Chen B, Zhou S, et al. Application of electrospun drug-loaded nanofibers in cancer therapy. Polymers. 2024;16:504. 10.3390/polym16040504. 38399882 10.3390/polym16040504 PMC10892891

[39] Shi W, Jiang Y, Wu T, Zhang Y, Li T. Advancements in drug-loaded hydrogel systems for bone defect repair. Regenerative Ther. 2024;25:174–85. 10.1016/j.reth.2023.12.010. 10.1016/j.reth.2023.12.010 PMC10789937 38230308

[40] Das S, Langbang L, Haque M, Belwal VK, Aguan K, Roy AS. Biocompatible silver nanoparticles: An investigation into their protein binding efficacies, anti-bacterial effects and cell cytotoxicity studies. J Pharmaceut Analysis. 2021;11:422–34. 10.1016/j.jpha.2020.12.003. 10.1016/j.jpha.2020.12.003 PMC8424387 34513118

[41] W.R. Rolim, M.T. Pelegrino, B.d.A. Lima, L.S. Ferraz, F.N. Costa, J.S. Bernardes, T. Rodigues, M. Brocchi, and A.B. Seabra, Green tea extract mediated biogenic synthesis of silver nanoparticles: Characterization, cytotoxicity evaluation and antibacterial activity. Applied Surface Science 463 (2019) 66–74.10.1016/j.apsusc.2018.08.203.

[42] H.A. Widatalla, L.F. Yassin, A.A. Alrasheid, S.A. Rahman Ahmed, M.O. Widdatallah, S.H. Eltilib, and A.A. Mohamed, Green synthesis of silver nanoparticles using green tea leaf extract, characterization and evaluation of antimicrobial activity. Nanoscale advances 4 (2022) 911–915.10.1039/d1na00509j. 10.1039/d1na00509j PMC9419201 36131825

[43] Ni Q, Zhu T, Wang W, Guo D, Li Y, Chen T, et al. Green synthesis of narrow-size silver nanoparticles using ginkgo biloba leaves: condition optimization, characterization, and antibacterial and cytotoxic activities. Int J Mol Sci. 2024;25:1913. 10.3390/ijms25031913. 38339192 10.3390/ijms25031913 PMC10856183

[44] Quiroz-Hernández JE, Kharissova OV, Aguirre-Arzola VE, Martinez-Avila GCG, Castillo-Velazquez U. Evaluation of the conditions for the synthesis of silver nanoparticles from orange peels and its antibacterial effect. Recent Patents Nanotechnol. 2020;14:250–8. 10.2174/1872210514666200414101014. 10.2174/1872210514666200414101014 32286951

[45] Aboul-Nasr MB, Yasien AA, Mohamed SS, Obiedallah M. Optimized biosynthesis of bioactive silver nanoparticle-kombucha cellulose nanocomposites for enhanced antimicrobial applications. Appl Microbiol Biotechnol. 2025;109:209. 10.1007/s00253-025-13585-0. 41021012 10.1007/s00253-025-13585-0 PMC12479647

[46] M. Behravan, A. Hossein Panahi, A. Naghizadeh, M. Ziaee, R. Mahdavi, and A. Mirzapour, Facile green synthesis of silver nanoparticles using Berberis vulgaris leaf and root aqueous extract and its antibacterial activity. International journal of biological macromolecules 124 (2019) 148–154.10.1016/j.ijbiomac.2018.11.101. 10.1016/j.ijbiomac.2018.11.101 30447360

[47] Zhang W, Jiang W. Antioxidant and antibacterial chitosan film with tea polyphenols-mediated green synthesis silver nanoparticle via a novel one-pot method. Int J Biol Macromol. 2020;155:1252–61. 10.1016/j.ijbiomac.2019.11.093. 31726160 10.1016/j.ijbiomac.2019.11.093

[48] Eker F, Akdaşçi E, Duman H, Bechelany M, Karav S. Green synthesis of silver nanoparticles using plant extracts: a comprehensive review of physicochemical properties and multifunctional applications. Int J Mol Sci. 2025;26:6222. 10.3390/ijms26136222. 40650001 10.3390/ijms26136222 PMC12250056

[49] Yin IX, Zhang J, Zhao IS, Mei ML, Li Q, Chu CH. The antibacterial mechanism of silver nanoparticles and its application in dentistry. Int J Nanomed. 2020;15:2555–62. 10.2147/ijn.S246764. 10.2147/IJN.S246764 PMC7174845 32368040

[50] Khorrami S, Zarrabi A, Khaleghi M, Danaei M, Mozafari MR. Selective cytotoxicity of green synthesized silver nanoparticles against the MCF-7 tumor cell line and their enhanced antioxidant and antimicrobial properties. Int J Nanomed. 2018;13:8013–24. 10.2147/ijn.S189295. 10.2147/IJN.S189295 PMC6267361 30568442

[51] RA Bapat, TV Chaubal, CP Joshi, PR Bapat, H Choudhury, M Pandey, B Gorain, P Kesharwani. An overview of application of silver nanoparticles for biomaterials in dentistry. Materials science & engineering. C, Materials for biological applications 91 (2018) 881–898.10.1016/j.msec.2018.05.069. 10.1016/j.msec.2018.05.069 30033323

[52] Chen X, Liu Z, Ma R, Gao S, Zheng W, Zhang F, et al. All-in-one nanounit embedded with biomimetic peptide for comprehensive caries repair. ACS nano. 2025;19:41684–703. 10.1021/acsnano.5c13970. 41326279 10.1021/acsnano.5c13970

[53] Liu R, Liu Y, Yi J, Fang Y, Guo Q, Cheng L, et al. Imbalance of oral microbiome homeostasis: the relationship between microbiota and the occurrence of dental caries. BMC Microbiol. 2025;25:46. 10.1186/s12866-025-03762-6. 39865249 10.1186/s12866-025-03762-6 PMC11770982

[54] Mummolo S, Nota A, Albani F, Marchetti E, Gatto R, Marzo G, et al. Salivary levels of Streptococcus mutans and Lactobacilli and other salivary indices in patients wearing clear aligners versus fixed orthodontic appliances: An observational study. PloS one. 2020;15:e0228798. 10.1371/journal.pone.0228798. 32330172 10.1371/journal.pone.0228798 PMC7182227

[55] Mummolo S, Quinzi V, Nota A, Marino C, Pittari L, Manenti RJ, et al. Metal versus fiberglass post-orthodontic retainers short-term effects on plaque index and microbial colonization: an observational study. Life (Basel, Switzerland). 2022;12:331. 10.3390/life12030331. 35330082 10.3390/life12030331 PMC8948786

[56] Lv C, Wang Z, Li Z, Shi X, Xiao M, Xu Y. Formation, architecture, and persistence of oral biofilms: recent scientific discoveries and new strategies for their regulation. Front Microbiol. 2025;16:1602962. 10.3389/fmicb.2025.1602962. 40703234 10.3389/fmicb.2025.1602962 PMC12285135

[57] Bowen WH, Burne RA, Wu H, Koo H. Oral biofilms: pathogens, matrix, and polymicrobial interactions in microenvironments. Trends Microbiol. 2018;26:229–42. 10.1016/j.tim.2017.09.008. 29097091 10.1016/j.tim.2017.09.008 PMC5834367

[58] Chen Y, Cao LM, Zhong NN, Li ZZ, Bu LL, Huo FY, et al. More than just aligning the teeth: Clear aligners with multifunctional prowess. Nano Res. 2024;17:7665–74. 10.1007/s12274-024-6631-4.

[59] Astasov-Frauenhoffer M, Göldi L, Rohr N, Worreth S, Dard E, Hünerfauth S, et al. Antimicrobial and mechanical assessment of cellulose-based thermoformable material for invisible dental braces with natural essential oils protecting from biofilm formation. Sci Rep. 2023;13:13428. 10.1038/s41598-023-39320-1. 37596293 10.1038/s41598-023-39320-1 PMC10439145

[60] Sun F, Hu W, Zhao Y, Li Y, Xu X, Li Y, et al. Invisible assassin coated on dental appliances for on-demand capturing and killing of cariogenic bacteria. Colloids and surfaces. B Biointerfaces. 2022;217:112696. 10.1016/j.colsurfb.2022.112696. 35834998 10.1016/j.colsurfb.2022.112696

[61] Park S, Kim HH, Yang SB, Moon JH, Ahn HW, Hong J. A polysaccharide-based antibacterial coating with improved durability for clear overlay appliances. ACS Appl Mater Interfaces. 2018;10:17714–21. 10.1021/acsami.8b04433. 29726672 10.1021/acsami.8b04433

[62] Wei R, Deng J, Guo X, Yang Y, Miao J, Liu A, et al. Construction of Zwitterionic coatings with lubricating and antiadhesive properties for invisible aligner applications. Macromol Rapid Commun. 2025;46:e2400234. 10.1002/marc.202400234. 38824415 10.1002/marc.202400234

[63] Zhang M, Liu X, Xie Y, Zhang Q, Zhang W, Jiang X, et al. Biological safe gold nanoparticle-modified dental aligner prevents the porphyromonas gingivalis biofilm formation. ACS Omega. 2020;5:18685–92. 10.1021/acsomega.0c01532. 32775870 10.1021/acsomega.0c01532 PMC7407536

[64] OY Ha, JM Oh, S Kim, Binder-Free TiO2-Cu 2-Cu composite powder coating for thermoformable orthodontic clear aligners. Chemical Engineering Journal. 2024;496.10.1016/j.cej.2024.153851.

[65] Shi Y, Zhang N, Liu J, Wang J, Shen S, Zhang J, et al. Preparation of nanocomposites for antibacterial orthodontic invisible appliance based on piezoelectric catalysis. Sensors. 2023;23:5336. 10.3390/s23115336. 37300063 10.3390/s23115336 PMC10256112

