# Supplementary material for: Femtosecond laser-induced micro/nanostructures loaded with silver nanoparticles on clear aligners prevent bacterial infection
Source: BMC Oral Health. 2026 Jun 23;26:1506. doi: 10.1186/s12903-026-08963-7 (PMC13483151; doi:10.1186/s12903-026-08963-7)
Supplement: Supplementary file 1 [file 12903_2026_8963_MOESM1_ESM.docx]

**Supplementary material**

1.The selected parameters (e.g., wavelength, pulse duration, power) were indeed optimized through preliminary experiments. As shown in the figure below, femtosecond laser processing at different powers resulted in distinct micro/nano-structures on the PETG surface. The influence of laser parameters on PETG surface structures is significant and warrants further exploration. At 20 W, the surface exhibited groove-like structures, which were ideal for drug loading due to their enhanced physical adsorption capacity. Thus, this power was chosen to balance structural integrity and functional performance.

Figure S1. SEM images of PETG surfaces processed at different laser powers.
